# Out-Door Life for Phthisis

**Published:** 1861-01

**Authors:** 


*Out-Door Life for Phthisis.*—Dr. Blake, of Sacramento, Cal., speaking of the advantages of the climate of California to consumptives, attributes much to the out-door life which it enables this class to lead, without exposure; and mentions the fa ct that Indian children domesticated, die in large numbers of phthisis.—*Am. Med. Times, Nov.* 10.

